# Pragmatics and the aims of language evolution

**DOI:** 10.3758/s13423-016-1061-2

**Published:** 2016-07-01

**Authors:** Thomas C. Scott-Phillips

**Affiliations:** 1 Department of Anthropology, Durham University, Durham, UK; 2 Evolutionary Anthropology Research Group, Department of Anthropology, Durham University, Dawson Building, South Road, Durham, DH1 3LE UK

**Keywords:** Pragmatics, Language evolution, Communication, Social cognition

## Abstract

Pragmatics has historically played a relatively peripheral role in language evolution research. This is a profound mistake. Here I describe how a pragmatic perspective can inform language evolution in the most fundamental way: by making clear what the natural objects of study are, and hence what the aims of the field should be.

## Strong and weak pragmatics, and their neglect in language evolution

**Fig. 1:**
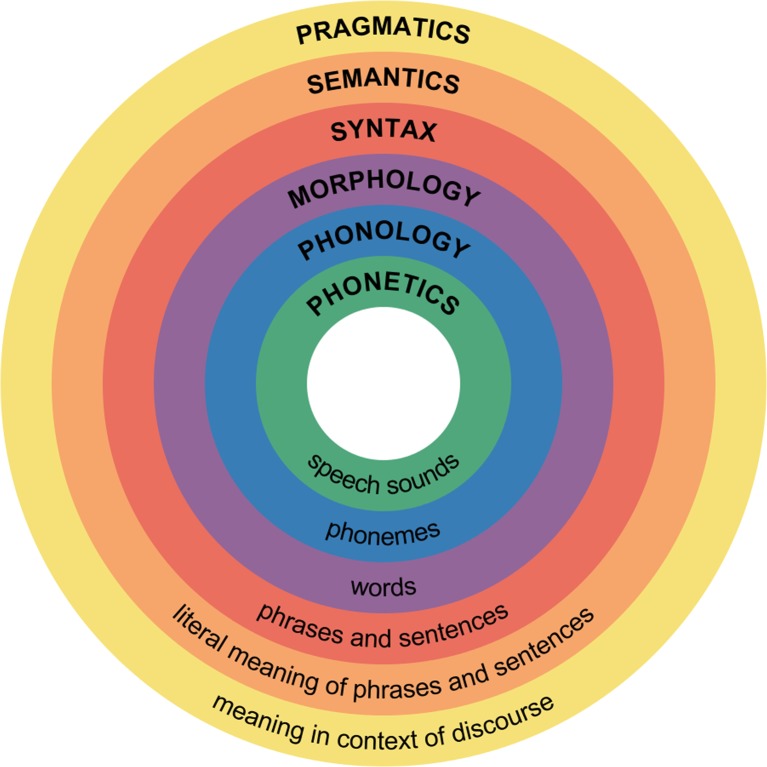
Major levels of linguistic structure, as classically understood.

For most of its history as an academic discipline, the principal intellectual concern of linguistics has been with the structure of the different levels of linguistic form. Figure 1 illustrates the different levels as classically understood (the image is from Wikimedia, where it is entitled “Major levels of linguistic structure”). For all but the outermost level, the items in question—sounds, phonemes, words, phrases, and literal meaning—are either discrete in character or can be treated as such. This discreteness makes quantitative and formal analysis more straightforward than it otherwise would be, and as such is a boon to scientific investigation. The sixth, outermost layer of Fig. 1—pragmatics—is where the linguistic rubber hits the communicative road, and this interface with the outside world brings with it a raft of issues that make any assumption of discreteness problematic, and as such complicate linguistic analysis. There is, for example, no simple and/or natural way to treat speaker meaning as a discrete object of enquiry.

Depending on one’s specific questions, it can be productive to abstract away from the complications that pragmatics brings with it. Indeed, this is sometimes the most scientifically appropriate thing to do. (All scientific investigation necessarily abstracts away from issues at other levels of analysis.) Many linguistic topics can be profitably pursued without any consideration of pragmatics. One consequence of this is that, despite common acknowledgment that it is an important dimension of language and language use, pragmatics is kept at the margins of linguistics as a discipline. In short, the fact that it is concerned with the boundary between language and the outside world is, ironically, a major reason why pragmatics is kept on the periphery of the discipline itself.

Even if unintended, this marginalization of pragmatics affects the direction of research in linguistics, and language evolution is a case in point. If you doubt this, turn to the index of the *Oxford Handbook of Language Evolution*, a book billed, accurately, as “a wide-ranging summation of work in all the disciplines involved [in language evolution]” (Tallerman & Gibson, 2012). There you will find 213 pages listed under “syntax” and related terms; 100 pages listed under “phonetics,” “phonology,” and related terms; but just eight pages under “pragmatics.” Alternatively, take a look at the lists of plenary speakers from the 11 Evolang conferences that have taken place up to 2016. You will find a relative dearth of pragmaticists. Two especially conspicuous omissions are Stephen Levinson and Dan Sperber, both high-profile pragmaticists who have written extensively about the origins and evolution of human communication and language. It is hard to escape the conclusion that pragmatics is of only peripheral concern to language evolution. This is, I believe, a profound mistake.

Consider a distinction between pragmatics in a “weak” and a “strong” sense. *Weak* pragmatics is simply context dependence—that is, the observation that the effects of different communicative behaviors depend, at least in part, on the local circumstances in which the behavior is produced. This type of “pragmatics” is widespread in the natural world. It may be a particularly salient feature of human communication, but it is certainly not uniquely human. It is not even unique to mammals. Weak pragmatics is implicit in Fig. 1, where it is effectively presented as a one further level of linguistic analysis, in addition to semantics, syntax, and the rest. It is also implicit in a significant proportion of research published in pragmatics itself.

**Fig. 2:**
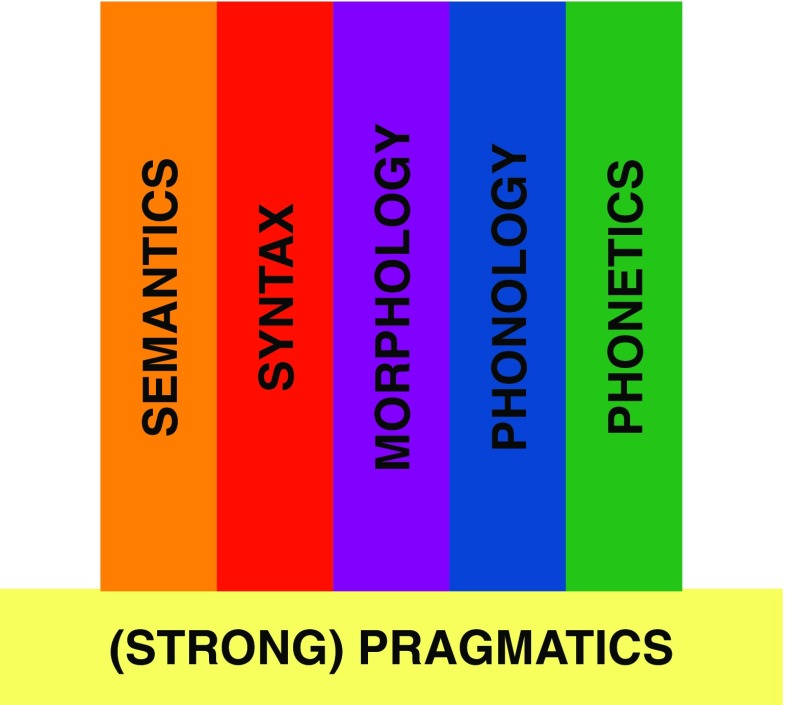
Pragmatics as the cognitive foundation for linguistic communication

In contrast, *strong* pragmatics is a capacity of mind, to communicate in a way that is fundamentally a matter of social cognition. More precisely, it is a capacity to communicate by expressing and recognizing intentions. This type of pragmatics commonly goes by the labels “Gricean communication” (after Paul Grice; see Grice, 1989, for a collection of his work on this topic) or “ostensive communication” (a label coined in Sperber & Wilson, 1995), and it is not only a further level of linguistic analysis. It is, rather, the social–cognitive basis of a type of communication that is not reducible to codes and context dependence (Carston, 2002a,b; Levinson, 2006; Origgi & Sperber, 2000; Scott-Phillips, 2014; Sperber & Wilson, 1995, 2002; Tomasello, 2008) and is likely to be uniquely human (Scott-Phillips, 2014, 2015a, b). With this in mind, Fig. 2 may be a more accurate depiction of the relationship between pragmatics and the other branches of linguistics.

For some subfields and for some questions, abstraction away from the messy realities of human social interaction, and toward the more abstract, idealized world of discrete linguistic items, can be a reasonable and profitable agenda. Pragmatics may have little to offer, say, phonetics or theoretical syntax. However, language evolution is not such a field. Once we recognize that linguistic communication is made possible by a capacity of mind for Gricean/ostensive communication, then understanding the evolution of this capacity becomes a critical issue. In fact, and as I shall now discuss, a pragmatic perspective not only highlights how important the evolution of ostensive/Gricean communication is for language evolution, it also clarifies what the field’s other key questions should be.

## A pragmatic perspective tells us what the key aims of language evolution should be

In *Relevance: Communication and Cognition* (1995), not to mention many publications since, Dan Sperber and Deirdre Wilson developed a detailed and compelling argument that linguistic communication exists on a continuum with other, nonlinguistic forms of communication, canonical examples of which include points, shrugs, and other nonverbal gestures. The overall category here is that of *ostensive communication*: communication that involves the expression and recognition of intentions—that is, “strong” pragmatics. The intentions involved are, specifically, communicative intentions (which can be roughly glossed as an intention to make apparent to the audience *that* one is trying to communicate) and informative intentions (which can be roughly glossed as an intention to make apparent to the audience *what* one is trying to communicate). Ostensive communication can be used for a great many communicative ends, but its expressivity is hugely increased by the addition of words, grammar, and the other communicative conventions that collectively comprise a language. I can make a request of others by ostensively pushing unchopped vegetables in their direction, but with specific conventions I can make requests about things remote in time and space. Linguistic communication is, then, a special case of ostensive communication, namely one in which expressivity is hugely increased by the existence of shared communicative conventions (Scott-Phillips, 2014).

If all this is correct, then already a pragmatic perspective has earned its keep, because it tells what two of the most central questions for language evolution should be. They are:

1. How and why did humans evolve ostensive communication/strong pragmatics?; and
2. How do collections of communicative conventions develop, and how and why do they evolve, culturally, to take the forms that they do?

Question 1 is about the biological evolution of ostensive communication, Question 2 about the cultural evolution of languages. My own answers to these questions are described at length in my book, *Speaking Our Minds* (2014; see Scott-Phillips, 2015c, for a précis).

The two questions above encompass many subquestions, and together they cover the majority of topics investigated in the name of language evolution (but see the next section for an exception). Regarding Question 1, the relevant issues include:

- The cognitive basis of nonhuman communication (What, exactly, are the cognitive mechanisms that make ostensive communication possible in the first place? Does any other species communicate in an ostensive way?)
- The selection pressures responsible for the evolution of ostensive communication (Sociality? Gossip? Teaching? Sex? Hunting?)
- The evolutionary stability of human communication (What processes maintain the stability of human communication? Why do the potential benefits of deception not cause the system to collapse?)

Regarding Question 2, the relevant issues include:

- The creation of novel sign systems (How do new systems get started? What role does iconicity play? How do we signal signalhood?)
- The factors that influence the direction of language change/evolution (In which directions do languages tend to evolve? Why? What factors play a causal role in this process? Which of these factors are shared with other species?)
- The nature of protolanguage (Analytic or synthetic? Gestural, vocal, or multimodal?)

These lists are not intended to be exhaustive. They are simply indicative lists of the sorts of issues that fall under each of the two main topic areas identified above. Note also that the domain of Question 2 is different from the domain of language change, which is concerned with changes from one established linguistic state to another (see Scott-Phillips & Kirby, 2010, for further discussion).

The importance of (strong) pragmatics does not stop here, with description of the important questions. A pragmatic perspective is also essential to answering these questions. Question 1 is fundamentally about pragmatics itself, and good answers to Question 2 will almost certainly include an important role for pragmatics, because the demands of expressing oneself in a comprehensible manner—that is, of pragmatics—are clearly a critical factor in the cultural evolution of languages.

Where, then, should future work be directed? In the case of Question 1, the further development of comparative approaches is clearly critical. As was mentioned above, pragmatics in the weaker sense of the term is biologically widespread; but what about pragmatics in the stronger sense of the term? One example of a relevant finding is the discovery that interactive turn-taking in communication takes place in all major primate clades (Levinson, 2016). However, there is much more to be done, and more focus should be directed to noncommunicative social cognition. Strong pragmatics is in the end a matter of mutually assisted social cognition: Signalers aim to affect the mental states of their audience, and the audience attempts to infer those intentions—and as such, comparisons between the social cognition of humans and other species is of high relevance to the evolution of ostensive communication (Scott-Phillips, 2015a, b; Tomasello, 2008).

Regarding Question 2, a key goal should be to link the study of the cultural evolution of languages with cognitive anthropology, one of the central concerns of which is how and why cultural items emerge and remain stable (see, e.g., Sperber, 1996). As was discussed above, languages are sets of cultural, communicative conventions, and language evolution is concerned with how these conventions develop the sort of properties that make them linguistic in the first place. As such, language evolution and cognitive anthropology each have much to offer the other—but this potential for mutually beneficial exchange has not yet been exploited in any substantial way.

## Beyond pragmatics: Language in its broad and narrow senses

Of course, some other important questions for language evolution are less fundamentally dependent on pragmatics than are those discussed above. The term *language* is not synonymous with either languages or communication. Instead, it is commonly—although not universally—used to describe whatever domain-specific capacities that humans have to acquire and use languages (Bolhuis et al., 2014). The qualifier “domain-specific” means that the capacity is functionally specific to a particular task or mechanism, and it is used to exclude a range of abilities that, although clearly relevant, are not specifically linguistic (the term *language faculty* is often used as a synonym for *language* in this sense). Perceptual abilities and memory are two obvious examples of cognitive abilities that are relevant to language use but not specifically linguistic. Others have made essentially the same distinction as this using the labels “faculty of language broad” (FLB: any aspect of biology or cognition that is employed in language acquisition and use) and “faculty of language narrow” (FLN: any *specifically linguistic* capacity that is employed in language acquisition and use; Hauser et al., 2002). Many researchers, especially biologists and psychologists, use *language* as being roughly synonymous with FLB; others—especially, but not only, linguists of the generative school—use it as synonymous with FLN. Either way, a critical question is what, if anything, is in FLN? “Certainly, humans are endowed with some sort of predisposition toward language learning. The substantive issue is whether a full description of that predisposition incorporates anything that entails specific contingent facts about natural languages” (Pullum & Scholz, 2002, p. 10). These are vexed issues, at the heart of the most prominent theoretical disputes in linguistics. There is no general consensus over the contents of FLN.

## Conclusion: Carving nature at its joints

In any scientific enterprise, much is to be gained from identification of the natural objects of study—that is, by “carving nature at its joints.” A pragmatic perspective helps to make clear what the natural objects of study are for language evolution. They are:

1. Human Gricean/ostensive communication (i.e., pragmatics in the “strong” sense of the word);
2. Languages (the sets of communicative conventions that enhance the expressive range of human ostensive communication);
3. Language/FLN (whatever domain-specific features of biology or cognition, if any, that humans have for the acquisition and use of languages); and
4. FLB (those aspects of biology or cognition that are employed in language acquisition and use, but are not specifically linguistic—e.g., memory, the speech apparatus).

Correspondingly, the aims of language evolution, as a field of study, should be to describe and explain the origins and evolution, whether biological or cultural, of each of these.
